# Supplementary material for: Mature oligodendrocytes bordering lesions limit demyelination and favor myelin repair via heparan sulfate production
Source: eLife. 2020 Jun 9;9:e51735. doi: 10.7554/eLife.51735 (PMC7308090; doi:10.7554/eLife.51735)
Supplement: Transparent reporting form [file elife-51735-transrepform.docx]

***eLife’s* transparent reporting form**

We encourage authors to provide detailed information *within their submission* to facilitate the interpretation and replication of experiments. Authors can upload supporting documentation to indicate the use of appropriate reporting guidelines for health-related research (see [EQUATOR Network](http://www.equator-network.org/%20)), life science research (see the [BioSharing Information Resource](https://biosharing.org/)), or the [ARRIVE guidelines](http://www.plosbiology.org/article/info:doi/10.1371/journal.pbio.1000412) for reporting work involving animal research. Where applicable, authors should refer to any relevant reporting standards documents in this form.

If you have any questions, please consult our Journal Policies and/or contact us: [editorial@elifesciences.org](mailto:editorial@elifesciences.org).

**Sample-size estimation**

- You should state whether an appropriate sample size was computed when the study was being designed
- You should state the statistical method of sample size computation and any required assumptions
- If no explicit power analysis was used, you should describe how you decided what sample (replicate) size (number) to use

Please outline where this information can be found within the submission (e.g., sections or figure legends), or explain why this information doesn’t apply to your submission:

-For mouse work : Sample-size estimation was not computed when the study was designed. We decided the sample size based on our previous studies with similar analyses.

-For human work, sample-size estimation was not completed. All available human tissue was used.

**Replicates**

- You should report how often each experiment was performed
- You should include a definition of biological versus technical replication
- The data obtained should be provided and sufficient information should be provided to indicate the number of independent biological and/or technical replicates
- If you encountered any outliers, you should describe how these were handled
- Criteria for exclusion/inclusion of data should be clearly stated
- High-throughput sequence data should be uploaded before submission, with a private link for reviewers provided (these are available from both GEO and ArrayExpress)

Details on the number of mice are indicated in the figure legends. Mice with no visible lesion on brain sections after surgery were not included in the study.

We provide a supplemental document showing mice distribution according to analyses performed. Cf « Mice reporting file-Macchi »

-Concerning human samples : Table 1 in the text indicates number of patients and the number of lesions and their classification.

The human post mortem experiment was performed once on all lesions/blocks/patients. One MS block was excluded because the researchers could not outline the lesions with certainty.

Please outline where this information can be found within the submission (e.g., sections or figure legends), or explain why this information doesn’t apply to your submission:

**Statistical reporting**

- Statistical analysis methods should be described and justified
- Raw data should be presented in figures whenever informative to do so (typically when N per group is less than 10)
- For each experiment, you should identify the statistical tests used, exact values of N, definitions of center, methods of multiple test correction, and dispersion and precision measures (e.g., mean, median, SD, SEM, confidence intervals; and, for the major substantive results, a measure of effect size (e.g., Pearson's r, Cohen's d)
- Report exact p-values wherever possible alongside the summary statistics and 95% confidence intervals. These should be reported for all key questions and not only when the p-value is less than 0.05.

Please outline where this information can be found within the submission (e.g., sections or figure legends), or explain why this information doesn’t apply to your submission:

Mice work : For each figures an excel files is attached including raw data and all statistical tests performed and reports. According to the small number of samples non-parametric statistical tests were performed using dedicated software for small size samples (StatXact and specific Excel Macro).

Concerning human data : An excel spreadsheet with the raw data and all statistical tests performed is attached. All statistical tests were done using GraphPad.

Tests used for statistical analysis and symbols to state significance are indicated in figure legends. Exact p values are stated in the text when differences between samples are mentioned.

(For large datasets, or papers with a very large number of statistical tests, you may upload a single table file with tests, Ns, etc., with reference to sections in the manuscript.)

**Group allocation**

- Indicate how samples were allocated into experimental groups (in the case of clinical studies, please specify allocation to treatment method); if randomization was used, please also state if restricted randomization was applied
- Indicate if masking was used during group allocation, data collection and/or data analysis

For Mouse work, animals were genotyped to form control versus mutant groups. In each group after surgery, randomly picked up mice were sacrificed at specific time points. Number of mice used, allocation in various groups according to analyses performed are fully described in a separate reporting file. Cf « Mice reporting file-Macchi ». Number of animals used (n) can be found in figure legends and in the result section.

Please outline where this information can be found within the submission (e.g., sections or figure legends), or explain why this information doesn’t apply to your submission:

**Additional data files (“source data”)**

- We encourage you to upload relevant additional data files, such as numerical data that are represented as a graph in a figure, or as a summary table
- Where provided, these should be in the most useful format, and they can be uploaded as “Source data” files linked to a main figure or table
- Include model definition files including the full list of parameters used
- Include code used for data analysis (e.g., R, MatLab)
- Avoid stating that data files are “available upon request”

Please indicate the figures or tables for which source data files have been provided:

One source data file per figure is included in the submitted version of the manuscript. Each file includes raw data, statistical analysis and reports.

One summary showing mice distribution according to analyses performed is provided « Mice reporting file-Macchi ».
